# Molecular Mutations and Their Cooccurrences in Cytogenetically Normal Acute Myeloid Leukemia

**DOI:** 10.1155/2017/6962379

**Published:** 2017-01-19

**Authors:** Mengning Wang, Chuanwei Yang, Le Zhang, Dale G. Schaar

**Affiliations:** ^1^Hematologic Malignancies and Stem Cell Transplant, Rutgers Cancer Institute of New Jersey, Robert Wood Johnson Medical School, Rutgers University, New Brunswick, NJ 08903, USA; ^2^Systems Biology, The University of Texas MD Anderson Cancer Center, Houston, TX, USA; ^3^Breast Medical Oncology, The University of Texas MD Anderson Cancer Center, Houston, TX, USA; ^4^College of Computer and Information Science, Southwest University, Chongqing, China

## Abstract

Adult acute myeloid leukemia (AML) clinically is a disparate disease that requires intensive treatments ranging from chemotherapy alone to allogeneic hematopoietic cell transplantation (allo-HCT). Historically, cytogenetic analysis has been a useful prognostic tool to classify patients into favorable, intermediate, and unfavorable prognostic risk groups. However, the intermediate-risk group, consisting predominantly of cytogenetically normal AML (CN-AML), itself exhibits diverse clinical outcomes and requires further characterization to allow for more optimal treatment decision-making. The recent advances in clinical genomics have led to the recategorization of CN-AML into favorable or unfavorable subgroups. The relapsing nature of AML is thought to be due to clonal heterogeneity that includes founder or driver mutations present in the leukemic stem cell population. In this article, we summarize the clinical outcomes of relevant molecular mutations and their cooccurrences in CN-AML, including* NPM1*,* FLT3*^ITD^,* DNMT3A*,* NRAS*,* TET2*,* RUNX1*,* MLL*^PTD^,* ASXL1*,* BCOR*,* PHF6*,* CEBPA*^biallelic^,* IDH1*,* IDH2*^R140^, and* IDH2*^R170^, with an emphasis on their relevance to the leukemic stem cell compartment. We have reviewed the available literature and TCGA AML databases (2013) to highlight the potential role of stem cell regulating factor mutations on outcome within newly defined AML molecular subgroups.

## 1. Introduction

Acute myeloid leukemia (AML) is the most common type of acute leukemia in adults. Although there have been landmark targeted therapies developed in other hematologic malignancies, such as imatinib for chronic myeloid leukemia and ibrutinib in chronic lymphocytic leukemia, induction chemotherapy for AML has not changed significantly for several decades [1, 2]. The notable exception being acute promyelocytic leukemia (APL) with the development of all-trans retinoic acid (ATRA) and arsenic trioxide (ATO) to overcome the block in myeloid differentiation due to the PML-RAR*α* fusion protein created by the translocation 15;17. Current AML induction therapies are successful in obtaining complete remission in approximately 75% of young (age < 60 years) de novo AML patients; however, most are destined to relapse. This clinical behavior suggested the presence of an underlying leukemic cell population responsible for the relapsing nature of AML despite the attainment of a complete remission through induction chemotherapy. The existence of leukemic stem cells in AML capable of recapitulating the disease was firmly established by transplant experiments utilizing immunocompromised mouse models two decades ago [3, 4]. To date, the knowledge derived from the discovery of leukemic AML stem cells is just beginning to be used in developing new therapeutic strategies and categorizing risk groups in patients. Patient outcomes in CN-AML, in particular, are widely diverse. The clinical validation of several additional molecular markers such as* FLT3*,* NPM1,* and* CEBPA* mutations has added a great deal to the prognostic stratification of CN-AML. Therefore, it is vital to build upon these advances by continuing to elucidate the biological characteristics and properties of leukemic stem cells and their regulating factors to assess their impact on AML treatment plans, the overarching question being what is the optimal consolidation strategy for each AML patient? Perhaps the incorporation of leukemia-stem cell mutations will add further clarity to which patients merit consolidation with allo-HCT and its attendant mortality and comorbidity and which AML patients can be safely managed with chemotherapy alone.

Historically, the French American British (FAB) classification system was used to subdivide AML into 8 subgroups (M0–M7) on a morphological basis [5]. The advent of cytogenetic studies enabled AML subtypes to be stratified into three risk groups, favorable, intermediate, and unfavorable risk. Using cytogenetics, clinicians could identify the favorable risk AML, such as the core binding factor leukemias [inv(16), t(16;16), and t(8;21)], and for this risk group, excellent long-term survival could be achieved with high dose cytarabine consolidation therapy alone. For patients with unfavorable risk, such as monosomies, 17p deletion, or complex abnormalities, there is a very low likelihood of cure with chemotherapy alone and consolidation with allo-HSCT is pursued if possible. Intermediate-risk patients include CN-AML, which comprises up to 40% to 50% of AML patients [6]. The clinical outcome of CN-AML patients varies widely and cannot be predicted based solely on cytogenetics.

The focus on improving our understanding of CN-AML prognosis and outcomes leads to the identification additional molecular markers of clinical significance. Mutations in nucleophosmin 1* (NPM1)*, fetal liver tyrosine kinase 3* (FLT3),* and CCAAT/enhancer binding protein *α (CEBPA)* have been shown to have clinically significant prognostic value [7]. The* FLT3* internal tandem duplication (ITD) mutation (*FLT3*^ITD^) is present in nearly one-third of AML cases and has been associated with adverse clinical outcomes including increased relapse risk and decreased overall survival (OS) [8].* FLT3*^ITD^ presence in CN-AML identified a subgroup of patients with more adverse outcome, particularly patients with a high mutant allelic frequency [9]. In addition to providing prognostic information, the* FLT3*^ITD^ is a therapeutic target as well. Sorafenib, a tyrosine kinase inhibitor targeting* FLT3*^ITD^ mutations, has been shown to increase event-free survival (EFS) and relapse-free survival (RFS) when added to both induction and consolidation therapies, although there was no OS benefit in the three years of follow-up in the newly diagnosed AML patients aged 60 years or younger [10].* NPM1* mutation has also been recently validated as a molecular marker of minimal residual disease (MRD) in* NPM1* mutation positive patients and the presence of MRD was shown to be the only independent prognostic factor for death in multivariate analysis [11].

Consolidative chemotherapy is utilized to eliminate residual leukemia cells and/or leukemic stem cells (LSC) after induction chemotherapy to reduce the chance of relapse. Risk of a relapse after induction chemotherapy and consolidation chemotherapy increases with the increased MRD, a condition which can be assessed by immunophenotypical detection of leukemia cells [12, 13]. Level of MRD correlates with the amount of leukemic stem cells and predicts outcome in AML [14–17]. Therefore, it is imperative to keep leukemic stem cells in mind when clinicians stratify patients for treatment purposes.

Advances in clinical genomics have identified an expanding array of recurrent molecular lesions in AML that will add layers of complexity to prognostic stratification needed to guide treatment and provide needed targets for new AML therapies. The evolving challenge is to incorporate these molecular abnormalities and their combinatorial effect on AML prognosis and in turn treatment strategies. The availability of this new AML data has created a requirement of a new classification system based on both cytogenetics and additional molecular lesions, which will be pivotal in establishing new clinical treatment guidelines. Furthermore, new classifications based on molecular abnormalities may help clinical trial design to develop targeted therapies to specific subgroups of AML patients. Recently a new AML classification system has been proposed by Papaemmanuil et al. [18]. Here the authors classified AML based on the presence of one or more driver mutations +/− other comutations into 11 different subgroups and correlated with clinical outcomes. This new classification system has provided insight regarding the effects of specific driver mutations and the additive effect seen when they are found in combination. In this paper, we summarize the significance of the most clinically relevant molecular mutations, cooccurrences of these mutations, and their functional role on leukemic stem cell population in relation to clinical outcomes based on this newly developed classification system.

## 2. The New AML Classification System

The proposed new AML classification system is based on a retrospective genomic analysis of 1540 AML patients in three prospective trials of the German-Austrian AML Study Group [18]. Patients received induction chemotherapy with idarubicin, cytarabine, and etoposide (ICE) with or without ATRA; high-risk patients were offered allo-HCT; intermediate-risk patients were offered a matched related donor allo-HCT, if a matched sibling was available; low risk patients received chemotherapy alone. The median follow-up period was 5.9 years.

In addition to cytogenetic analyses, 111 candidate driver genes were sequenced and 5234 somatic driver mutations were identified across 76 genes or genomic regions. Nearly all AML patients (96%) had at least one mutation and 86% patients were found to have two or more mutations. Statistical analysis of comutation patterns was utilized to define 11 mutually exclusive AML subtypes including three novel genetic subgroups that have not been described in the World Health Organization (WHO) classification in 2008 [19]. These novel subgroups are, namely, (1) AML with mutations in genes encoding chromatin, RNA-splicing regulators, or both (18% of patients); (2) AML with* TP53* mutations, chromosomal aneuploidies, or both (13%); and (3) AML with* IDH2*^R172^ mutations (1%). Many of the mutations used to define the novel subgroups involve genes which have roles in stem cell functions. Of note, only 48% of patients were classifiable based on the current WHO guidelines, whereas 80% of patients could be allocated into this novel classification system. Only 8% of patients had either no detected driver mutations (4%) or ≥2 genomic subgroups (4%).

Clinical outcomes, such as OS of AML patients with genetic mutations, were found to be significantly altered by the presence or absence of other driver mutations as has been described by others [20]. For instance,* NPM1*-mutated AML, as the largest subgroup in this novel classification, had variable clinical outcomes influenced by the presence of concurrent mutations such as* FLT3*,* DNMT3A*,* NRAS*,* IDH*,* PTPN11*, or chromatin-spliceosome mutations. We focused on the clinical outcomes of the most significant single or concurrent molecular mutations based on this novel classification (Table 1) and the significance of concurrent or mutually exclusive alterations in genes of interest (Table 2). We did not include the effect of traditional cytogenetic abnormalities in this study.

## 3. AML with* NPM1* Mutation

Nucleophosmin is a protein encoded by the* NPM1* gene in humans. Nucleophosmin has multiple functions in various processes including histone chaperones, ribosome biogenesis and transport, genomic stability and DNA repair, control of centrosome duplications, and regulation of the ARF-p53 tumor suppressor pathway [21]. All these functions have a part in leukemic stem cell self-renewal and limited differentiation. Indeed, stem cell/progenitor cell surface marker CD34+ cells from* NPM1*-mutated AML patients are able to recapitulate leukemia in immunodeficient mice [22]. Alteration of the* NPM1* gene was found to be present at a high frequency in AML patients, ranging from 25% to 53% in all AML and 46% to 67% in CN-AML.* NPM1*-mutated AML consists of 27% of all AML and therefore forms the largest subgroup in this novel classification [18]. The identification of* NPM1* mutation in AML is important for both disease prognosis and the subsequent treatment decision-making regarding consolidation with chemotherapy alone or an allo-HCT treatment. Furthermore, a recent study demonstrated the importance of MRD analysis in* NPM1*-mutated AML [11]. Since high MRD correlates positively with high stem cell frequency in AML [16], persistence of* NPM1*-mutated transcripts in blood was associated with a greater risk of relapse after 3 years of follow-up.

AML with* NPM1* mutation is a clinically heterogeneous group likely due to the prevalence of concurrent mutations: 54%* DNMT3A*, 39%* FLT3*^ITD^, 19%* NRAS*, 16%* TET2,* and 15%* PTPN11*.* NPM1* is usually a secondary or downstream mutation, whereas mutations in* DNMT3A*,* ASXL1*,* IDH1*/*2*, and* TET2* occur very early during clonal evolution but are typically not sufficient to cause leukemia on their own. Therefore, the analysis of comutation patterns in this group has become crucial in predicting disease prognosis.

### 3.1. *NPM1* and* FLT3*^ITD^

*FLT3* ^ITD^ represents one of the most frequent genetic alterations with a 20% frequency in adult AML, 28–34% in cytogenetically normal AML [23], and 39% in* NPM1*-mutated AML [18].* FLT3*^ITD^ activates STAT5 to maintain survival of leukemic stem cell population in AML [24]. It was not a surprise that* FLT3*^ITD^-positive AML patients had higher relapse incidence and lower DFS [25, 26] as well as OS [27]. These observations have validated* FLT3* as a therapeutic target in AML and* FLT3* inhibitors have shown promising results when combined with standard therapy [10, 28].

With regard to* NPM1* and* FLT3*^ITD^, several studies have shown that AML with* NPM1* mutation, but without* FLT3*^ITD^ mutation, is associated with significantly better OS and EFS [29–32]; one study demonstrated that* NPM1* mutation with or without* FLT3*^ITD^ was only favorable in achieving complete remission but was associated with a high relapse rate with no OS and EFS benefits [33]. The German-Austrian AML Study Group conducted a study to evaluate genetic mutations and clinical outcomes in 872 adults younger than 60 years of age and again demonstrated that* NPM1* mutation without* FLT3*^ITD^ was associated with lower risk of relapse and death [34]. The majority of these earlier studies showed that* NPM1* mutation without* FLT3*^ITD^ is associated with better clinical outcomes, and allo-HST conferred no benefit in this patient group [34] similar to the core binding-factor leukemia patient group [35]. It is apparent that all these studies pointed to a worse clinical outcome when* NPM1 *mutation and* FLT3*^ITD^ mutation coexisted in AML. However, the most recent study argued against* NPM1* and* FLT3*^ITD^ mutations being the sole determinants in AML prognosis, and another mutation,* DNMT3A*, must also be taken into consideration in the decision-making process of the treatment of* NPM1*-mutated AML [18].

### 3.2. *NPM1* and* DNMT3A*

**Figure 1:**
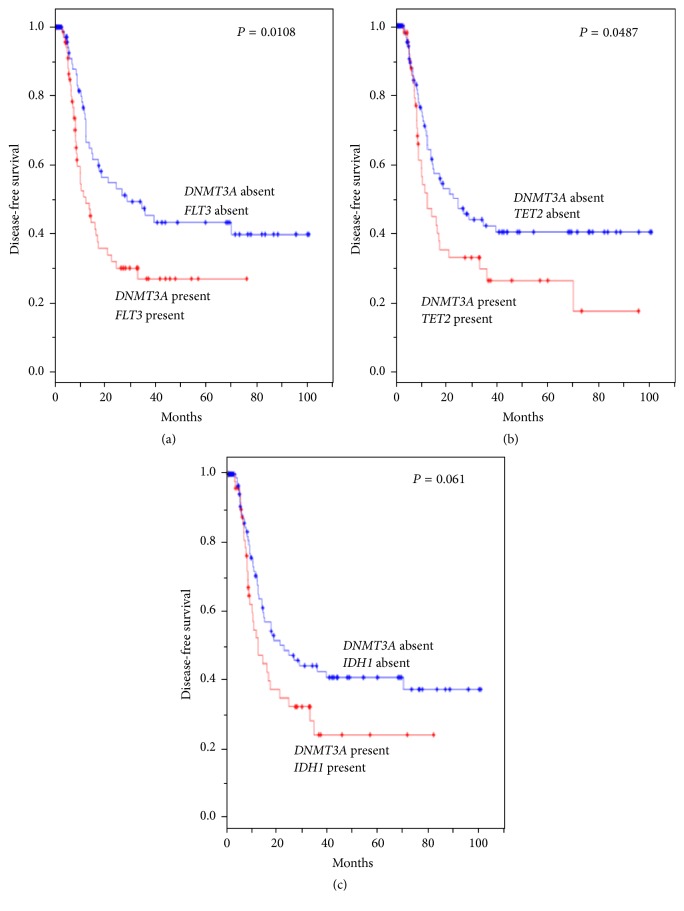
Kaplan-Meier curves for disease-free survival according to the presence or absence of the specific gene alterations. Gene alterations include mutations, deletions, fusions, and gene amplifications. All the alterations for* IDH1* are mutations. Over 95% of the alterations are mutations for* DNMT3A*,* TET2,* and* FLT3*. The rest of the alterations are multiple alterations for* DNMT3A* and* TET2* and deletions for* FLT3*. Database used for analysis is TCGA, NEJM 2013 [94]. The cBio Cancer Genomics Portal was used for the analysis [95] (http://cbioportal.org).

*DNMT3A* (DNA methyltransferase 3A) is an enzyme that catalyzes the transfer of methyl groups to specific CpG structures in DNA and hence plays an essential role in DNA methylation and gene silencing regulatory processes [36].* DNMT3A* is important in normal hematopoietic stem cell differentiation and self-renewal [37] and its mutation produces a reservoir of preleukemic stem cells which can evolve into AML [38].* DNMT3A* mutations were found in 22.1% of all AML and 33.7% of AML with intermediate-risk cytogenetic profile and were independently associated with a poor outcome regardless of age [39].* DNMT3A* mutations tend to cooccur with* FLT3*,* TET2*, or* IDH1* in AML (Table 2). The combination of* DNMT3A* mutation with* FLT3*,* TET2*, or* IDH1* tends to have an adverse effect on disease-free survival in AML compared to wild-type group (Figure 1). Interestingly, hypomethylating agents, such as decitabine and 5-azacitidine, have a higher clinical remission rate in* DNMT3A*-mutated AML [40].

Patients with* DNMT3A*,* TET2*,* ASXL1*,* PHF6*, or* MLL*^PTD^ mutations who were in the WHO intermediate group had an adverse outcome compared to those with other genotypes [27].* DNMT3A* mutation was found to be an adverse prognostic factor in cytogenetically normal AML with mutated* NPM1* without* FLT3*^ITD^ in terms of OS [41]. However, this finding was not confirmed by the most recent study [18]. Instead, it was reported that patients with both* NPM1* and* DNMT3A* mutations but without* FLT3*^ITD^ showed much better outcomes than those with* FLT3*^ITD^. Therefore, triple-mutated AML (*NPM1*/*DNMT3A*/*FLT3*^ITD^) yields the worst prognosis and the consolidation with allo-HCT should be considered, although prospective study is needed to confirm these results.

### 3.3. *NPM1* and* NRAS*^G12/13^

*NRAS* belongs to the RAS GTPase family of genes. It plays important roles in the regulation of proliferation, differentiation, and apoptosis in AML and is a fairly common mutation in AML ranging from 11% to 30% [42].* NRAS* mutation consists of 19% of* NPM1*-mutated AML [18]. The prognostic impact of* NRAS* mutation has been reported to be insignificant for OS, EFS, and disease-free survival (DFS) [42]. However more recently mutations in* NPM1* and* DNMT3A* in the presence of* NRAS*^G12/13^ in AML patients were associated with a more favorable outcome [18].

### 3.4. *NPM1* and* TET2*

*TET2* (ten-eleven translocation) protein is an epigenetic modifier that converts methylcytosine to 5-hydroxymethylcytosine and plays a role in DNA methylation and myelopoiesis. Normal expression and function of* TET2* are essential in maintaining the hematopoietic stem cell (HSC) pool and in controlling HSC differentiation [43]. Studies using conditional knockout mouse models have revealed that complete loss of* TET2 (TET2*^−/−^) or* TET2* haplodeficiency* (TET2*^+/−^) impaired hematopoietic stem cell differentiation, held cells in a more immature state, and initiated aberrant hematopoiesis both in vitro and in vivo [44–46].* TET2* expression is tightly regulated by the master stem cell transcription factors* Oct4* and* Sox2* [44].* TET2* mutations are present in 5–25% of adult AML cases, with the highest frequency in the elderly [47, 48].* TET2* mutations are significantly correlated with* NPM1* (16%) in this most recent study [18] and were found to be mutually exclusive with* MLL*^PTD^ [48] and* IDH1*/*2* mutations [48, 49].* TET2* mutation resulted in a lower complete remission rate, shorter EFS and DFS in patients with mutated* NPM1* without* FLT3*^ITD^ [47], and shorter OS in patients with mutated* NPM1* without* FLT3*^ITD^ [47, 50] and with mutated* NPM1* [50]. However,* TET2* mutations were also reported to have no impact on the clinical outcomes of de novo AML [48, 49], CN-AML [49, 50], and CN-AML with mutated* NPM1* or* CEBPA* without* FLT3*^ITD^ [41]. The full clinical impact of* TET2* mutations has yet to be fully understood.

### 3.5. *NPM1* and* IDH1/IDH2*^R140^

*IDH1* and* IDH2* (isocitrate dehydrogenases 1 and 2) are enzymes that catalyze the interconversion of isocitrate and alpha-ketoglutarate and appear to play an epigenetic role in histone and possibly DNA methylation.* IDH1* or* IDH2* mutations confer a hypermethylation phenotype in leukemia and inhibit hematopoietic stem cell differentiation [51]. These phenotypic characteristics are shared by* TET2* loss-of-function mutations [46]. The most common* IDH1* mutation is in the arginine residue at position 132 (*IDH1*^R132^), occurring in 6–9% of adult AML, while* IDH2* mutations occur in 9–19%, predominantly* IDH2*^R140^ in 8–12% [18, 27, 52–54].* IDH1* and* IDH2* mutations are mutually exclusive in AML. Furthermore,* IDH1* and* IDH2*^R140^ are strongly associated with* NPM1* mutations [18].* IDH1* [53, 54] and* IDH2* [53] mutations have been reported to carry an unfavorable prognosis with regard to survival in normal karyotype AML lacking* NPM1* and* FLT3*^ITD^ mutations. In patients with cooccurring* DNMT3A* and* IDH2*^R140^ mutations, the OS was significantly poorer than those with wild-type or a single mutation [18].

An earlier study demonstrated that the* IDH1* and* IDH2* mutations constitute poor prognostic factors in cytogenetically normal AML with* NPM1* mutation without* FLT3*^ITD^ [55]. In some other studies, however, patients with mutated* IDH1* or* IDH2*^R140^ had good prognoses for OS in AML patients with the* NPM1* mutation without* FLT3*^ITD^ [27, 52], and it was further concluded that the favorable effect of* NPM1* mutations was restricted to patients with cooccurring* NPM1* and* IDH1* or* IDH2*^R140^ mutations [27].

## 4. AML with Mutated Chromatin, RNA-Splicing Genes, or Both

This chromatin-spliceosome group is the second largest subgroup in this new classification [18]. This is also an extremely heterogeneous group, consisting of genes regulating RNA splicing (*SRSF2*,* SF3B1*,* U2AF1*, and* ZRSR2*), chromatin (*ASXL1*,* STAG2*,* BCOR*,* MLL*^PTD^,* EZH2*, and* PHF6*), and transcription* (RUNX1)*. Functional proteins encoded by these genes have functions in hematopoietic stem cell self-renewal and differentiation. Using the European LeukemiaNet (ELN) guidelines, the majority (84%) of the patients in this new chromatin-spliceosome group would be classified as having intermediate prognostic risk. However, this new subgroup demonstrated resistance to induction chemotherapy and inferior long-term outcomes [18] suggesting a reclassification of AML patients with chromatin-spliceosome mutations as an adverse prognostic group. Nearly all of the genetic mutations in this subgroup have been previously reported to be adverse prognostic markers.

### 4.1. *RUNX1*

The* RUNX1* (runt-related transcription factor 1, formerly known as AML1) gene encodes the alpha subunit of the core binding factor involved in transcription and is required for definitive hematopoiesis [56].* RUNX1* protein also plays an essential role in mesenchymal stem cell proliferation and promotes cell survival in AML [57, 58].* RUNX1* mutations are present in 5% to 18% of AML [59–62]. They are associated with* ASXL1* [59],* MLL*^PTD^ [62], and* IDH1*/*IDH2* mutations [62] and are essentially mutually exclusive of* NPM1* mutations [59, 62].* RUNX1* mutations were found to be associated with resistance to chemotherapy, inferior DFS, EFS [59, 61, 62], and OS [59–62]. More importantly,* RUNX1* mutations were deemed to be an independent prognostic marker for shorter EFS in multivariable analysis [62]. An explorative subgroup analysis demonstrated that* RUNX1*-mutated AML patients benefited from allo-HSC in terms of RFS [62].

### 4.2. *MLL*^PTD^

The* MLL *(Mixed Lineage Leukemia) gene, located on chromosome 11q23, is frequently involved in translocations that recur in AML and have been classified into an individual subgroup, AML with* MLL* fusion genes, t(x;11)(x;23) [18]. MLL fusion proteins are capable of transforming normal hematopoietic stem cells into malignant leukemic stem cells [63]. A* MLL* partial tandem duplication (*MLL*^PTD^), the result of a tandem duplication of an internal portion of the* MLL* gene that spans either exons 2 to 6 or exons 2 to 8, is present in approximately 10% of CN-AML [64, 65].* MLL*^PTD^ mutation has been identified as a poor prognostic factor for EFS [60, 66, 67] and OS [27, 41, 60, 67]. Furthermore, it is clear that the OS was shortened when the intermediate-risk group patients had mutated* MLL*^PTD^ regardless of the presence of* FLT3*^ITD^ [27].

### 4.3. *ASXL1*

The* ASXL1* (additional sex combs like-1) gene is a human homologue of the* Drosophila* additional sex combs* (Asx)* gene, which is highly conserved across multiple species. The* ASXL1* protein functions in both epigenetic activation and repression of gene transcription [68–70]. Its regulation of histone modification affects hematopoietic stem cell pool maintenance and its loss causes severe defects in HSC development [71, 72].* ASXL1* mutations are more common in the aberrant karyotypes, the elderly, and MDS-associated and secondary AML [73–77], while 9–12% of* ASXL1* mutations are detected in cytogenetically normal AML [75–77].* NPM1* and* ASXL1* mutations appear to be mutually exclusive [76–78]. Several studies have shown that AML patients with* ASXL1* mutations had worse outcomes when compared to those without these mutations [27, 60, 75]. Specifically in CN-AML,* ASXL1* mutations were associated with inferior complete remission, DFS, OS, and EFS [77].

### 4.4. *BCOR*

The* BCOR* (*BCL6* corepressor) gene is located on chromosome X and encodes a nuclear protein that is a key transcriptional regulator of hematopoiesis [79]. Studies demonstrated that normal* BCOR* retains hematopoietic stem cells in a quiescent, undifferentiated state and loss-of-function* BCOR* mutations result in enhanced HSC cell proliferation and differentiation [80].* BCOR* mutations occur in 3.8% of CN-AML, and* DNMT3A* mutations are detected in 43.5% of these patients.* BCOR* mutations tend to be associated with an inferior EFS and OS [81].

### 4.5. *PHF6*

The* PHF6* (plant homeodomain finger 6) gene, also located on chromosome X, plays a key role in chromatin remodeling [82].* PHF6* mutations are found in approximately 3% of adult AML and confer worse OS in intermediate-risk patients that are* FLT3*^ITD^ negative [27].

## 5. AML with* CEBPA*^**biallelic**^ Mutation

The* CEBPA* gene is located on chromosome 19 band q13.11 and encodes a 42 kDa size protein that is a member of the basic region leucine zipper transcription factor family [83]. Normal function of* CEBPA* is crucial in maintaining adult hematopoietic stem cell in a quiescent state and* CEBPA* gene knockout in mice results in impaired HSC differentiation [84]. The CEBPA protein is expressed in myelomonocytic cells and is critical for neutrophil development [83]. Mutated* CEBPA* regulates Sox4 expression which affects self-renewal of leukemic stem cells [85]. The frequency of* CEBPA* mutations is reported to range from 7% to 22% in patients with AML [86] and 15% to 18% in CN-AML [87, 88]. About two-thirds of* CEBPA*-mutated patients are biallelic-mutated (*CEBPA*^biallelic^), and the remaining one-third carry a single mutation (*CEBPA*^monoallelic^). In a meta-analysis of 10 clinical studies covering 6219 patients,* CEBPA*^biallelic^ mutation was found to be associated with favorable clinical outcomes with regard to EFS and OS in patients with AML or CN-AML; conversely, no significant difference was found between* CEBPA*^monoallelic^ mutation and wild-type* CEBPA* in patients with AML or CN-AML [86]. In a long-term follow-up study (median follow-up time of 9.8 years), patients with* CEBPA*^biallelic^ mutations showed longer OS, longer relapse-free survival, and a lower cumulative incidence of relapse compared to those with* CEBPA*^monoallelic^ mutation. The ten-year OS rate for patients ≤60 years and with* CEBPA*^biallelic^ mutation was 81%.* CEBPA*^biallelic^-mutated AML was associated with* TET2* mutation in 34% of the patients, and the combination resulted in significantly worse OS, whereas it was associated with* GATA2* (GATA binding protein 2, a transcription factor) mutation, found in 21% of* CEBPA*^biallelic^-mutated AML, resulting in improved OS [89].

## 6. AML with* IDH2*^**R172**^ Mutation

*IDH2* ^R172^ is a distinct* IDH2* mutation that occurs in AML with a frequency of 1–3% [18, 52, 90]. Unlike* IDH2*^R140^ that significantly correlates with* NPM1* mutation,* IDH2*^R172^ is generally not associated with other molecular mutations. The gene expression and DNA methylation profiles of* IDH2*^R172^-mutated AML differ from those of other IDH mutations and lead to more severe aberrations in metabolic activity [91, 92]. Thus,* IDH2*^R172^ mutation has been defined as a unique subgroup in the new classification scheme [18]. In previous studies,* IDH2*^R172^ mutation was associated with a higher relapse rate and lower OS that were comparable with those of the adverse-risk cytogenetics patients [52, 90]. In this most recent study, however, the presence of* IDH2*^R172^ mutation was associated with a favorable prognosis with regard to OS, similar to patients with* NPM1*-mutated AML [18].

## 7. Conclusions and Future Directions

The progress in AML risk stratification using next-generation sequencing technologies over the past decade has been truly remarkable. As an example, the identification of* NPM1*- or* CEBPA*^biallelic^-mutated CN-AML to have favorable risk has significantly impacted the clinical management of these patient groups. The novel AML classification system proposed by Papaemmanuil et al. has been especially valuable in organizing the growing array of AML mutations in terms of additive effects on prognosis. As an example, the subgroup “AML with* NPM1* mutation” is the largest subgroup and has a large number of comutations. The different comutation combinations do not have a strictly additive effect on clinical outcomes indicating further risk stratification in this group is necessary. Conversely, the subgroup “AML with mutated chromatin, RNA-splicing genes, or both” is more consistent as an adverse prognostic group, at least among the five genetic mutations we reviewed here:* RUNX1*,* MLL*^PTD^,* ASXL1, BCOR, and PHF6*. A central theme of this adverse-risk group is that the majority of these genes have roles in maintaining normal HSC quiescence by their epigenetic regulation and their mutations result in transformation of HSC into leukemic stem cells. These malignant stem cells, in turn, are thought to be the wellspring of leukemic cell expansion, likely directly responsible for the relapsing nature of AML. Of note, mutations in epigenetic modifiers or regulators such as* DNMT3A*,* TET2*, and* IDH1*/*2* alter normal HSC quiescent state and prime HSC to a preleukemic state [93]. These epigenetic factors function as stem cell regulators and impact DFS (Figure 1). This observation may be true beyond AML and therapies targeting epigenetic modifiers or stem cell regulating factors may hold promise in improving disease-free survival of patients with other hematologic malignancies. These observations of recurrent AML mutations and comutation patterns await validation in larger prospective clinical trials. Regardless, this new classification strategy is an important step forward in understanding the molecular complexity of AML and has the potential to yield many new therapeutic targets to be exploited to someday eradicate this aggressive disease.

## Figures and Tables

**Table 1:** Molecular classification of CN-AML and clinical outcomes.

AML with *NPM1* mutation	
*NPM1*^*∗*^	Better EFS and OS [29–32]; better CRR, high RR with EFS or OS benefits [33]; better OS [34]
*NPM1*/*DNMT3A*	Worse OS [41]
*NPM1*/*DNMT3A*/*FLT3*^ITD^	Worse OS [18]
*NPM1*/*DNMT3A*/*NRAS*^G12/13^	Better OS [18]
*NPM1*/*TET2*^*∗*^	Worse CRR, EFS, DFS, and OS [50]; no impact on outcomes [41]
*NPM1*/*IDH1*^*∗*^ or *NPM1*/*IDH2*^R140*∗*^	Worse OS [55]; better OS [27, 52]

AML with mutated chromatin, RNA-splicing genes, or both	
*RUNX1*	Worse EFS, DFS [59, 61, 62]; worse OS [59–62]
*MLL*^PTD^	Worse EFS [60, 66, 67]; worse OS [27, 41, 60, 67]
*ASXL1*	Worse outcomes [27, 60, 75]; worse CR, EFS, DFS, and OS [77]
*BCOR*	Worse EFS and OS [81]
*PHF6*^*∗*^	Worse OS [27]
AML with *CEBPA*^biallelic^ mutation	
*CEBPA*^biallelic^	Better EFS and OS [86]
*CEBPA*^biallelic^/*TET2*	Worse OS [89]
*CEBPA*^biallelic^/*GATA2*	Better OS [89]

AML with *IDH2*^R172^ mutation	Worse RR and OS [52, 90]; better OS [18]

EFS: event-free survival; OS: overall survival; CRR: complete remission rate; RR: relapse rate; DFS: disease-free survival.

^*∗*^Without *FLT3*^ITD^.

**Table 2:** Cooccurrent or mutually exclusive alterations and their significance in genes of interests.

Gene A	Gene B	*P* value	Odds ratio	Association	
*ASXL1*	*IDH2*	0.003	2.241	Tendency towards cooccurrence	Significant
*IDH1*	*DNMT3A*	0.005	1.266	Tendency towards cooccurrence	Significant
*TET2*	*DNMT3A*	0.01	1.156	Tendency towards cooccurrence	Significant
*DNMT3A*	*FLT3*	0.035	0.713	Tendency towards cooccurrence	Significant
*KMT2A*	*DNMT3A*	0.042	−1.169	Tendency towards mutual exclusivity	Significant
*IDH2*	*KMT2A*	0.056	1.052	Tendency towards cooccurrence	Marginal

Database used for analysis is TCGA, NEJM 2013 [94]. The database contains all 166 complete tumors of AML. Query was performed on 10 genes which include *ASXL1*, *BCOR*, *TET2*, *IDH1*, *IDH2*, *RUNX1*, *PHF6*, *KMT2A (MLL)*, *DNMT3A*, and *FLT3*. The query results contain 23 gene pairs with mutually exclusive alterations (1 significant) and 22 gene pairs with cooccurrent alterations (4 significant, 1 marginal).

## References

[1] Preisler H., Davis R. B., Kirshner J. (1987). Comparison of three remission induction regimens and two postinduction strategies for the treatment of acute nonlymphocytic leukemia: a cancer and leukemic group B study. *Blood*.

[2] Wiernik P. H., Banks P. L. C., Case D. C. (1992). Cytarabine plus idarubicin or daunorubicin as induction and consolidation therapy for previously untreated adult patients with acute myeloid leukemia. *Blood*.

[3] Bonnet D., Dick J. E. (1997). Human acute myeloid leukemia is organized as a hierarchy that originates from a primitive hematopoietic cell. *Nature Medicine*.

[4] Lapidot T., Sirard C., Vormoor J. (1994). A cell initiating human acute myeloid leukaemia after transplantation into SCID mice. *Nature*.

[5] Bennett J. M., Catovsky D., Daniel M. T. (1985). Proposed revised criteria for the classification of acute myeloid leukemia: a report of the French-American-British Cooperative Group. *Annals of Internal Medicine*.

[6] Heim S., Mitelman F. (2015). *Cancer Cytogenetics: Chromosomal and Molecular Genetic Aberrations of Tumor Cells*.

[7] Betz B. L., Hess J. L. (2010). Acute myeloid leukemia diagnosis in the 21st century. *Archives of Pathology and Laboratory Medicine*.

[8] Kottaridis P. D., Gale R. E., Frew M. E. (2001). The presence of a FLT3 internal tandem duplication in patients with acute myeloid leukemia (AML) adds important prognostic information to cytogenetic risk group and response to the first cycle of chemotherapy: analysis of 854 patients from the United Kingdom Medical Research Council AML 10 and 12 trials. *Blood*.

[9] Santos F. P. S., Jones D., Qiao W. (2011). Prognostic value of FLT3 mutations among different cytogenetic subgroups in acute myeloid leukemia. *Cancer*.

[10] Röllig C., Serve H., Hüttmann A. (2015). Addition of sorafenib versus placebo to standard therapy in patients aged 60 years or younger with newly diagnosed acute myeloid leukaemia (SORAML): a multicentre, phase 2, randomised controlled trial. *The Lancet Oncology*.

[11] Ivey A., Hills R. K., Simpson M. A. (2016). Assessment of minimal residual disease in standard-risk AML. *New England Journal of Medicine*.

[12] Kern W., Voskova D., Schoch C., Hiddemann W., Schnittger S., Haferlach T. (2004). Determination of relapse risk based on assessment of minimal residual disease during complete remission by multiparameter flow cytometry in unselected patients with acute myeloid leukemia. *Blood*.

[13] Feller N., van der Pol M. A., van Stijn A. (2004). MRD parameters using immunophenotypic detection methods are highly reliable in predicting survival in acute myeloid leukaemia. *Leukemia*.

[14] Gentles A. J., Plevritis S. K., Majeti R., Alizadeh A. A. (2010). Association of a leukemic stem cell gene expression signature with clinical outcomes in acute myeloid leukemia. *JAMA - Journal of the American Medical Association*.

[15] van Rhenen A., Moshaver B., Kelder A. (2007). Aberrant marker expression patterns on the CD34+CD38− stem cell compartment in acute myeloid leukemia allows to distinguish the malignant from the normal stem cell compartment both at diagnosis and in remission. *Leukemia*.

[16] van Rhenen A., Feller N., Kelder A. (2005). High stem cell frequency in acute myeloid leukemia at diagnosis predicts high minimal residual disease and poor survival. *Clinical Cancer Research*.

[17] Venditti A., Buccisano F., Del Poeta G. (2000). Level of minimal residual disease after consolidation therapy predicts outcome in acute myeloid leukemia. *Blood*.

[18] Papaemmanuil E., Gerstung M., Bullinger L. (2016). Genomic classification and prognosis in acute myeloid leukemia. *New England Journal of Medicine*.

[19] Vardiman J. W., Thiele J., Arber D. A. (2009). The 2008 revision of the World Health Organization (WHO) classification of myeloid neoplasms and acute leukemia: rationale and important changes. *Blood*.

[20] Grimwade D., Ivey A., Huntly B. J. P. (2016). Molecular landscape of acute myeloid leukemia in younger adults and its clinical relevance. *Blood*.

[21] Lindström M. S. (2011). NPM1/B23: a multifunctional chaperone in ribosome biogenesis and chromatin remodeling. *Biochemistry Research International*.

[22] Martelli M. P., Pettirossi V., Thiede C. (2010). CD34^+^ cells from AML with mutated *NPM1* harbor cytoplasmic mutated nucleophosmin and generate leukemia in immunocompromised mice. *Blood*.

[23] Marcucci G., Haferlach T., Döhner H. (2011). Molecular genetics of adult acute myeloid leukemia: prognostic and therapeutic implications. *Journal of Clinical Oncology*.

[24] Yoshimoto G., Miyamoto T., Jabbarzadeh-Tabrizi S. (2009). FLT3-ITD up-regulates MCL-1 to promote survival of stem cells in acute myeloid leukemia via FLT3-ITD-specific STAT5 activation. *Blood*.

[25] Thiede C., Steudel C., Mohr B. (2002). Analysis of FLT3-activating mutations in 979 patients with acute myelogenous leukemia: association with FAB subtypes and identification of subgroups with poor prognosis. *Blood*.

[26] Brunet S., Labopin M., Esteve J. (2012). Impact of FLT3 internal tandem duplication on the outcome of related and unrelated hematopoietic transplantation for adult acute myeloid leukemia in first remission: a retrospective analysis. *Journal of Clinical Oncology*.

[27] Patel J. P., Gönen M., Figueroa M. E. (2012). Prognostic relevance of integrated genetic profiling in acute myeloid leukemia. *New England Journal of Medicine*.

[28] Hassanein M., Almahayni M. H., Ahmed S. O., Gaballa S., El Fakih R. (2016). FLT3 inhibitors for treating acute myeloid leukemia. *Clinical Lymphoma Myeloma and Leukemia*.

[29] Schnittger S., Schoch C., Kern W. (2005). Nucleophosmin gene mutations are predictors of favorable prognosis in acute myelogenous leukemia with a normal karyotype. *Blood*.

[30] Döhner K., Schlenk R. F., Habdank M. (2005). Mutant nucleophosmin (NPM1) predicts favorable prognosis in younger adults with acute myeloid leukemia and normal cytogenetics: interaction with other gene mutations. *Blood*.

[31] Verhaak R. G. W., Goudswaard C. S., Van Putten W. (2005). Mutations in nucleophosmin (NPM1) in acute myeloid leukemia (AML): Association with other gene abnormalities and previously established gene expression signatures and their favorable prognostic significance. *Blood*.

[32] Thiede C., Koch S., Creutzig E. (2006). Prevalence and prognostic impact of NPM1 mutations in 1485 adult patients with acute myeloid leukemia (AML). *Blood*.

[33] Suzuki T., Kiyoi H., Ozeki K. (2005). Clinical characteristics and prognostic implications of NPM1 mutations in acute myeloid leukemia. *Blood*.

[34] Schlenk R. F., Döhner K., Krauter J. (2008). Mutations and treatment outcome in cytogenetically normal acute myeloid leukemia. *New England Journal of Medicine*.

[35] Schlenk R. F., Benner A., Krauter J. (2004). Individual patient data–based meta-analysis of patients aged 16 to 60 years with core binding factor acute myeloid leukemia: a survey of the German Acute Myeloid Leukemia Intergroup. *Journal of Clinical Oncology*.

[36] Bestor T. H. (2000). The DNA methyltransferases of mammals. *Human Molecular Genetics*.

[37] Challen G. A., Sun D., Jeong M. (2012). Dnmt3a is essential for hematopoietic stem cell differentiation. *Nature Genetics*.

[38] Shlush L. I., Zandi S., Mitchell A. (2014). Identification of pre-leukaemic haematopoietic stem cells in acute leukaemia. *Nature*.

[39] Ley T. J., Ding L., Walter M. J. (2010). DNMT3A mutations in acute myeloid leukemia. *New England Journal of Medicine*.

[40] Metzeler K. H., Walker A., Geyer S. (2012). DNMT3A mutations and response to the hypomethylating agent decitabine in acute myeloid leukemia. *Leukemia*.

[41] Kihara R., Nagata Y., Kiyoi H. (2014). Comprehensive analysis of genetic alterations and their prognostic impacts in adult acute myeloid leukemia patients. *Leukemia*.

[42] Bacher U., Haferlach T., Schoch C., Kern W., Schnittger S. (2006). Implications of NRAS mutations in AML: a study of 2502 patients. *Blood*.

[43] Ko M., Huang Y., Jankowska A. M. (2010). Impaired hydroxylation of 5-methylcytosine in myeloid cancers with mutant TET2. *Nature*.

[44] Koh K. P., Yabuuchi A., Rao S. (2011). Tet1 and Tet2 regulate 5-hydroxymethylcytosine production and cell lineage specification in mouse embryonic stem cells. *Cell Stem Cell*.

[45] Moran-Crusio K., Reavie L., Shih A. (2011). Tet2 loss leads to increased hematopoietic stem cell self-renewal and myeloid transformation. *Cancer Cell*.

[46] Cimmino L., Abdel-Wahab O., Levine R. L., Aifantis I. (2011). TET family proteins and their role in stem cell differentiation and transformation. *Cell Stem Cell*.

[47] Metzeler K. H., Maharry K., Radmacher M. D. (2011). TET2 mutations improve the new European LeukemiaNet risk classification of acute myeloid leukemia: a cancer and leukemia group B study. *Journal of Clinical Oncology*.

[48] Damm F., Markus B., Thol F. (2014). TET2 mutations in cytogenetically normal acute myeloid leukemia: clinical implications and evolutionary patterns. *Genes Chromosomes and Cancer*.

[49] Gaidzik V. I., Paschka P., Späth D. (2012). TET2 mutations in Acute Myeloid Leukemia (AML): results from a comprehensive genetic and clinical analysis of the AML study group. *Journal of Clinical Oncology*.

[50] Tian X., Xu Y., Yin J. (2014). TET2 gene mutation is unfavorable prognostic factor in cytogenetically normal acute myeloid leukemia patients with NPM1^+^ and FLT3-ITD^−^ mutations. *International Journal of Hematology*.

[51] Figueroa M. E., Abdel-Wahab O., Lu C. (2010). Leukemic IDH1 and IDH2 mutations result in a hypermethylation phenotype, disrupt TET2 function, and impair hematopoietic differentiation. *Cancer Cell*.

[52] Green C. L., Evans C. M., Zhao L. (2011). The prognostic significance of IDH2 mutations in AML depends on the location of the mutation. *Blood*.

[53] Yamaguchi S., Iwanaga E., Tokunaga K. (2014). IDH1 and IDH2 mutations confer an adverse effect in patients with acute myeloid leukemia lacking the NPM1 mutation. *European Journal of Haematology*.

[54] Abbas S., Lugthart S., Kavelaars F. G. (2010). Acquired mutations in the genes encoding IDH1 and IDH2 both are recurrent aberrations in acute myeloid leukemia: prevalence and prognostic value. *Blood*.

[55] Paschka P., Schlenk R. F., Gaidzik V. I. (2010). IDH1 and IDH2 mutations are frequent genetic alterations in acute myeloid leukemia and confer adverse prognosis in cytogenetically normal acute myeloid leukemia with NPM1 mutation without FLT3 internal tandem duplication. *Journal of Clinical Oncology*.

[56] Okuda T., Van Deursen J., Hiebert S. W., Grosveld G., Downing J. R. (1996). AML1, the target of multiple chromosomal translocations in human leukemia, is essential for normal fetal liver hematopoiesis. *Cell*.

[57] Goyama S., Schibler J., Cunningham L. (2013). Transcription factor RUNX1 promotes survival of acute myeloid leukemia cells. *The Journal of Clinical Investigation*.

[58] Kim W., Barron D. A., San Martin R. (2014). RUNX1 is essential for mesenchymal stem cell proliferation and myofibroblast differentiation. *Proceedings of the National Academy of Sciences of the United States of America*.

[59] Mendler J. H., Maharry K., Radmacher M. D. (2012). RUNX1 mutations are associated with poor outcome in younger and older patients with cytogenetically normal acute myeloid leukemia and with distinct gene and microRNA expression signatures. *Journal of Clinical Oncology*.

[60] Grossmann V., Schnittger S., Kohlmann A. (2012). A novel hierarchical prognostic model of AML solely based on molecular mutations. *Blood*.

[61] Tang J.-L., Hou H.-A., Chen C.-Y. (2009). AML1/RUNX1 mutations in 470 adult patients with de novo acute myeloid leukemia: prognostic implication and interaction with other gene alterations. *Blood*.

[62] Gaidzik V. I., Bullinger L., Schlenk R. F. (2011). RUNX1 mutations in acute myeloid leukemia: results from a comprehensive genetic and clinical analysis from the AML study group. *Journal of Clinical Oncology*.

[63] Krivtsov A. V., Armstrong S. A. (2007). MLL translocations, histone modifications and leukaemia stem-cell development. *Nature Reviews Cancer*.

[64] Caligiuri M. A., Strout M. P., Lawrence D. (1998). Rearrangement of ALL1 (MLL) in acute myeloid leukemia with normal cytogenetics. *Cancer Research*.

[65] Caligiuri M. A., Strout M. P., Schichman S. A. (1996). Partial tandem duplication of ALL1 as a recurrent molecular defect in acute myeloid leukemia with trisomy 11. *Cancer Research*.

[66] Döhner K., Tobis K., Ulrich R. (2002). Prognostic significance of partial tandem duplications of the MLL gene in adult patients 16 to 60 years old with acute myeloid leukemia and normal cytogenetics: a study of the Acute Myeloid Leukemia Study Group Ulm. *Journal of Clinical Oncology*.

[67] Shen Y., Zhu Y.-M., Fan X. (2011). Gene mutation patterns and their prognostic impact in a cohort of 1185 patients with acute myeloid leukemia. *Blood*.

[68] Cho Y.-S., Kim E.-J., Park U.-H., Sin H.-S., Um S.-J. (2006). Additional sex comb-like 1 (ASXL1), in cooperation with SRC-1, acts as a ligand-dependent coactivator for retinoic acid receptor. *Journal of Biological Chemistry*.

[69] Scheuermann J. C., De Ayala Alonso A. G., Oktaba K. (2010). Histone H2A deubiquitinase activity of the Polycomb repressive complex PR-DUB. *Nature*.

[70] Boultwood J., Perry J., Pellagatti A. (2010). Frequent mutation of the polycomb-associated gene ASXL1 in the myelodysplastic syndromes and in acute myeloid leukemia. *Leukemia*.

[71] Abdel-Wahab O., Gao J., Adli M. (2013). Deletion of Asxl1 results in myelodysplasia and severe developmental defects in vivo. *Journal of Experimental Medicine*.

[72] O'Brien E. C., Prideaux S., Chevassut T. (2014). The epigenetic landscape of acute myeloid leukemia. *Advances in Hematology*.

[73] Devillier R., Gelsi-Boyer V., Brecqueville M. (2012). Acute myeloid leukemia with myelodysplasia-related changes are characterized by a specific molecular pattern with high frequency of *ASXL1*mutations. *American Journal of Hematology*.

[74] Fernandez-Mercado M., Yip B. H., Pellagatti A. (2012). Mutation patterns of 16 genes in primary and secondary acute myeloid leukemia (AML) with normal cytogenetics. *PLoS ONE*.

[75] Chou W.-C., Huang H.-H., Hou H.-A. (2010). Distinct clinical and biological features of de novo acute myeloid leukemia with additional sex comb-like 1 (ASXL1) mutations. *Blood*.

[76] Schnittger S., Eder C., Jeromin S. (2013). ASXL1 exon 12 mutations are frequent in AML with intermediate risk karyotype and are independently associated with an adverse outcome. *Leukemia*.

[77] Metzeler K. H., Becker H., Maharry K. (2011). ASXL1 mutations identify a high-risk subgroup of older patients with primary cytogenetically normal AML within the ELN Favorable genetic category. *Blood*.

[78] Carbuccia N., Trouplin V., Gelsi-Boyer V. (2010). Mutual exclusion of ASXL1 and NPM1 mutations in a series of acute myeloid leukemias. *Leukemia*.

[79] Huynh K. D., Fischle W., Verdin E., Bardwell V. J. (2000). BCoR, a novel corepressor involved in BCL-6 repression. *Genes and Development*.

[80] Cao Q., Gearhart M. D., Gery S. (2016). BCOR regulates myeloid cell proliferation and differentiation. *Leukemia*.

[81] Grossmann V., Tiacci E., Holmes A. B. (2011). Whole-exome sequencing identifies somatic mutations of BCOR in acute myeloid leukemia with normal karyotype. *Blood*.

[82] Todd M. A. M., Ivanochko D., Picketts D. J. (2015). Phf6 degrees of separation: the multifaceted roles of a chromatin adaptor protein. *Genes*.

[83] Zhang D.-E., Zhang P., Wang N.-D., Hetherington C. J., Darlington G. J., Tenen D. G. (1997). Absence of granulocyte colony-stimulating factor signaling and neutrophil development in CCAAT enhancer binding protein *α*-deficient mice. *Proceedings of the National Academy of Sciences of the United States of America*.

[84] Tenen D. G. (2014). Myeloid differentiation and the leukemia-initiating cell. *Leukemia Supplements*.

[85] Zhang H., Alberich-Jorda M., Amabile G. (2013). Sox4 is a key oncogenic target in C/EBP*α* mutant acute myeloid leukemia. *Cancer Cell*.

[86] Li H.-Y., Deng D.-H., Huang Y. (2015). Favorable prognosis of biallelic *CEBPA* gene mutations in acute myeloid leukemia patients: a meta-analysis. *European Journal of Haematology*.

[87] Fröhling S., Schlenk R. F., Stolze I. (2004). CEBPA mutations in younger adults with acute myeloid leukemia and normal cytogenetics: prognostic relevance and analysis of cooperating mutations. *Journal of Clinical Oncology*.

[88] Bienz M., Ludwig M., Mueller B. U. (2005). Risk assessment in patients with acute myeloid leukemia and a normal karyotype. *Clinical Cancer Research*.

[89] Grossmann V., Haferlach C., Nadarajah N. (2013). *CEBPA*double-mutated acute myeloid leukaemia harbours concomitant molecular mutations in 76·8% of cases with *TET2*and *GATA2*alterations impacting prognosis. *British Journal of Haematology*.

[90] Boissel N., Nibourel O., Renneville A. (2010). Prognostic impact of isocitrate dehydrogenase enzyme isoforms 1 and 2 mutations in acute myeloid leukemia: A Study by the Acute Leukemia French Association Group. *Journal of Clinical Oncology*.

[91] Marcucci G., Maharry K., Wu Y.-Z. (2010). IDH1 and IDH2 gene mutations identify novel molecular subsets within de novo cytogenetically normal acute myeloid leukemia: a cancer and leukemia group B study. *Journal of Clinical Oncology*.

[92] Chen C., Liu Y., Lu C. (2013). Cancer-associated IDH2 mutants drive an acute myeloid leukemia that is susceptible to Brd4 inhibition. *Genes & Development*.

[93] Corces-Zimmerman M. R., Hong W.-J., Weissman I. L., Medeiros B. C., Majeti R. (2014). Preleukemic mutations in human acute myeloid leukemia affect epigenetic regulators and persist in remission. *Proceedings of the National Academy of Sciences of the United States of America*.

[94] The Cancer Genome Atlas Research Network (2013). Genomic and epigenomic landscapes of adult de novo acute myeloid leukemia. *The New England Journal of Medicine*.

[95] Cerami E., Gao J., Dogrusoz U. (2012). The cBio cancer genomics portal: an open platform for exploring multidimensional cancer genomics data. *Cancer Discovery*.

